# Tau vulnerability in entorhinal substructure and tau's proximity to vasculature

**DOI:** 10.1002/alz70855_102022

**Published:** 2025-12-23

**Authors:** Jean Augustinack, Josué Llamas Rodríguez, Smriti Chadha, Matthew P. Frosch, André J.W. van der Kouwe

**Affiliations:** ^1^ Harvard Medical School, Charlestown, MA, USA; ^2^ Massachusetts General Hospital, Charlestown, MA, USA

## Abstract

**Background:**

Healthy memory function relies on an intact entorhinal‐hippocampal circuit. Hippocampal volume is and has been a staple in neuroimaging to structurally assess memory status. However, in Alzheimer's disease, the presence of phosphorylated tau (*p*‐tau) in hippocampus occurs late in disease progression after memory impairment has begun. The entorhinal cortex (EC) represents an early target to evaluate, given that *p*‐tau forms earlier in EC than hippocampus and EC is the site responsible for the conversion from healthy cognitive aging to AD. Evaluating the entorhinal cortex, as a whole, is not sensitive or early enough in disease pathogenesis. To study this, we evaluated *p*‐tau in EC by substructure, as well as other neighboring neurodegenerative pathologies (e.g. TDP‐43) and even tau's proximity to vasculature.

**Method:**

We obtained 18 postmortem brain samples to assess the selective vulnerability of particular subfields within EC. Medial temporal lobe tissue blocks were fixed, excised, cryoprotected, sectioned, and immunostained for *p*‐tau CP13. We applied a subfield framework dividing the entorhinal cortex into eight subfields. Multiple anterior‐posterior sections were staged for tau severity. We evaluated tau burden semi‐quantitatively by subfield, building on Braak's staging by adding tau burden density. The neuronal (EC II) cell type was also indexed. We acquired also postmortem MRI at 100 micron isotropic resolution on a subset of samples (*n* = 12), which enabled us to visualize intracortical vessels in 3D. We manually labeled intracortical vasculature on the high‐resolution MRI were reconstructed to show vessel density in the entire EC.

**Result:**

Our results show that the posterior subfields, ECs and ECL, represent the selectively vulnerable subfields at early stage (preclinical BBI and BBII) before cognitive impairment. Spearman's test showed a positive correlation between posterior EC and higher tau semi‐quantitative score (Spearman's r = 0.956). We created multiple individual anterior to posterior matrices for the tau histopathology per subfield to show selective vulnerability at a precise level. The vessels in EC were most dense in regions/subfields that showed the highest tau burden.

**Conclusion:**

The tau validated matrices, reconstructed vessel maps and particular neuronal populations provide perspective of the neuronal environment and the contribution to vulnerability.

